# Innovation and Discovery: A 30-Year Journey in Advancing Cancer Care

**DOI:** 10.3390/curroncol31040156

**Published:** 2024-04-08

**Authors:** Shahid Ahmed

**Affiliations:** 1 Division of Oncology, University of Saskatchewan, Saskatoon, SK S7N5E5, Canada; shahid.ahmed@saskcancer.ca; 2 Saskatchewan Cancer Agency, Saskatoon Cancer Center, Saskatoon, SK S7N5H5, Canada

Since the inaugural issue of *Current Oncology* was published 30 years ago, we have witnessed significant advancements in cancer research and care. The approval of rituximab by the US Food and Drug Administration for refractory CD20-positive B-cell non-Hodgkin lymphoma in November 1997 heralded a new era of targeted therapy in cancer therapeutics and is considered to be a major milestone in precision medicine [1]. This was soon followed by a report of the effectiveness of trastuzumab, a humanized anti-HER2 receptor monoclonal antibody, in HER2-positive metastatic breast cancer, which was subsequently found to be beneficial in reducing cancer recurrence and mortality in early-stage breast cancer [2,3]. This was a major innovation that changed the treatment landscape of breast cancer and inspired the development of small molecules, drug–antibody conjugates, and other targeted therapies for the management of breast cancer and other malignancies. At the same time, we saw the remarkable efficacy of imatinib against chronic myelogenous leukemia (CML), targeting the BCR-ABL fusion gene, which subsequently showed efficacy in gastrointestinal stromal tumors [4]. Imatinib altered the natural history of CML, leading to its fast-track approval.

Over the subsequent decades, breakthroughs in immunotherapy have dramatically changed the treatment landscape for many cancers. After the approval of the first human cancer vaccine treatment in 2010, collaborative research has demonstrated the tumor-agnostic effect of immune checkpoint inhibitors in many cancers [5]. More recently, cell-based immunotherapy using chimeric antigen receptor (CAR)-T cell therapy represents a major advance in oncology [6]. In parallel with these therapeutic advancements, the mapping of the human genome was a key step that enhanced our understanding of cancer genetics immensely and accelerated the evolution of targeted therapy and precision medicine in cancer treatment [7].

Notably, these innovations in cancer research span the Cancer Care continuum. For example, the introduction of human papilloma virus (HPV) vaccine is considered a crucial step in preventing cervical and other HPV-related cancers [8]. Chemoprevention trials for breast cancer in women and prostate cancer in men yielded positive results [9,10]. Similarly, lung cancer screening with low-dose CT scans in individuals at high risk for lung cancer, the leading cause of cancer-related death, has resulted in low mortality rates [11]. Technological advancements have also played a crucial role in advancing cancer research and care during this time. Innovations such as clustered regularly interspaced short palindromic repeats (CRISPR) gene editing, artificial intelligence, tele-health, cryo-electron microscopy, and robotic surgery have revolutionized our approaches to cancer research, diagnosis, and treatment. These examples only highlight a few of the key achievements the global cancer community has made in improving patient care.

As we mark the 30th anniversary of *Current Oncology*, it is important to recognize the key role it has played in the advancement of cancer knowledge and patient care. Through the collaborative efforts of the editorial staff, authors, and reviewers, *Current Oncology* has established itself as the reputable peer-review clinical oncology journal within the oncology community. Since the publication of its first issue, it has provided a dedicated platform for the oncology community for the dissemination of key translational, clinical, health services, and health economics research, contributing to innovation and revolutionary changes in cancer care. It has published more than 3800 papers with over 32,000 citations, including original articles, reviews, commentaries, clinical practice guidelines, and consensus reports that have gone through a vigorous peer-review process, contributing to the expansion of new knowledge and innovative approaches in cancer care. With the use of an open-access platform, *Current Oncology* has facilitated the global dissemination of knowledge, connecting researchers, and fostering collaboration and new discoveries.

Furthermore, *Current Oncology* has published Special Issues focusing on important themes in cancer research to highlight key areas in cancer research, promote interdisciplinary collaboration, and disseminate new knowledge and education in emerging areas among the cancer community. These achievements would not have been possible without the dedication and hard work of the editorial staff, including over 300 academic editors and more than 5000 reviewers, along with more than 13,000 authors whose contributions are vital to the success of *Current Oncology*. Their commitment to advancing cancer research and improving patient outcomes is the foundation of *Current Oncology*. To recognize the dedication and valuable contributions of researchers, young investigators, and reviewers, *Current Oncology* offers several awards. These include the Young Investigator Award, the Travel Award, and the Outstanding Reviewer Award, aimed at appreciating efforts and commitment of the cancer research community in promoting new knowledge.

As we celebrate three decades of progress in cancer research, we must recognize the need for ongoing innovation and discovery in cancer care. Currently, cancer remains a major global burden, with an estimated 20 million new cases diagnosed and approximately 10 million deaths in 2022 [12]. Lung cancer continues to be the leading cause of cancer death. Millions of women are diagnosed with breast cancer, and despite treatment advancement, many still develop recurrent disease and die from advanced breast cancer. Cervical cancer, though preventable, still claims around 350,000 women’s lives annually. The five-year survival rate for pancreatic cancer is only around 10%, and colorectal cancer cases are rising rapidly in young adults. Moreover, disparity in access to cancer care continues to be a major challenge [13]. The present challenges in cancer care underscore the ongoing need for global efforts and collaborative research across the cancer care continuum, from prevention and early diagnosis to treatment, survivorship care, and supportive care, along with a continued need for basic science and translational research. It is imperative that these collective efforts are aimed at further understanding cancer biology and the tumor micro-environment to help to identify more effective targets, overcome drug resistance, reduce treatment toxicities, and facilitate personalized medicine, while also identifying more effective preventative and screening strategies, reducing inequity in care, and addressing the physical and mental well-being of cancer survivors.

*Current Oncology* remains committed to the dissemination of innovation and discovery in cancer research. This Special Issue, marking the 30th anniversary of *Current Oncology*, aims to advance cancer care through the publication of high-quality research papers, reflecting our commitment to fostering collective efforts in cancer research.
